# Cardiorespiratory Fitness May Protect Memory for Poorer Sleepers

**DOI:** 10.3389/fpsyg.2022.793875

**Published:** 2022-02-17

**Authors:** Tara Kuhn, Jennifer Heisz

**Affiliations:** Department of Kinesiology, McMaster University, Hamilton, ON, Canada

**Keywords:** sleep, memory, aging – old age – seniors, physical activity, exercise, cardiorespiratory fitness, cognition

## Abstract

**Objectives:**

Physical activity has been shown to protect executive functions against the deleterious effects of poorer sleep among older adults (OA); however, it is unknown whether memory is protected too, and if this relationship differs by age. The present study investigated the relationship between cardiorespiratory fitness, sleep, and memory in both older and young adults (YA).

**Methods:**

This observational study recruited 26 OA (70.7 ± 2.8 years) and 35 YA (21.0 ± 3.1 years). Participants completed the Rockport 1-mile walk test to evaluate cardiorespiratory fitness. Participants wore an actigraph for 1 week to measure habitual sleep and returned for a second visit to perform the memory tests. The interaction between cardiorespiratory fitness and sleep to predict memory was assessed separately in OA and YA.

**Results:**

In OA, cardiorespiratory fitness significantly moderated the relationship between memory and sleep quality, specifically number of nighttime awakenings, sleep efficiency, and wake after sleep onset. Further analyses reveal that a high number of nighttime awakenings and low sleep efficiency significantly predicted worse memory performance in the low fit OA, but high fit OA. Notably, every nighttime awakening was associated with a nearly 4% decrease in memory in low fit OA, but not high fit OA. Wake after sleep onset did not significantly predict memory in either fitness group. No interaction was found when looking at sleep duration or self-report sleep quality in OA and no significant interactions were observed between fitness, sleep, and memory in YA.

**Conclusion:**

Overall, the results suggest that cardiorespiratory fitness may act as a protective buffer for memory in OA with poor sleep quality. These same was not true for YA suggesting that the protective effects of cardiorespiratory fitness on sleep-related memory impairments may be age specific.

## Introduction

Sleep is vital for good health. Experts have set nightly sleep requirements of 7–9 h for adults between 18 and 65 years old and 7–8 h of sleep for older adults (OA) over the age of 65 years old (Hirshkowitz et al., 2015). Yet nearly 1 in 3 adults fail to achieve these recommendations (Chaput et al., 2017). To make matters worse, many OA may be in bed for the recommended amount of time but struggle to fall asleep and stay asleep (Chaput et al., 2017). Such poor sleep quality is related to poor cognition (Blackwell et al., 2011; Lim et al., 2012), accelerated cognitive decline (Bruce and Aloia, 2006; Altena et al., 2010) and dementia (Beaulieu-Bonneau and Hudon, 2009; Lim et al., 2013; Sterniczuk et al., 2013). Unfortunately, with aging, sleep quality naturally declines. Compared to younger adults (YA), OA sleep less, have more difficulty initiating and maintaining sleep, and spend less time in slow wave sleep (SWS) – sleep’s deepest and most restorative stage (Ohayon et al., 2004; Moraes et al., 2014; Mander et al., 2017).

Physical activity may be an effective way to promote sleep and counteract the deleterious effects of poor sleep on cognition. Both acute and chronic physical activity improve sleep quality (Kubitz et al., 1996; Kredlow et al., 2015) and cognition (Colcombe and Kramer, 2003; Chang et al., 2010; Middleton et al., 2010; Roig et al., 2013; Loprinzi et al., 2018). However, less is known about whether physical activity protects poor sleepers against their additional cognitive deficits. To date, only two studies have examined the interplay between exercise, sleep, and cognition in healthy humans and both of those studies focused on executive functioning (EF), a subset of cognitive processes including working memory, inhibitory control, and cognitive flexibility (Diamond, 2013). One study found that sleep efficiency, but not sleep duration, mediated the relationship between physical activity and EF in YA and OA (Wilckens et al., 2018). These results suggest physical activity may enhance sleep quality, which in turn, may improve EF (Wilckens et al., 2018). The other study (Lambiase et al., 2014), examined the relationship between sleep and EF using a subset of data from the Healthy Women Study. They found that poor sleepers who were also *low active* had the poorest cognitive flexibility. However, the participants who were *high active* performed similarly well regardless of how poor they slept. These results are promising and suggest that physical activity may help protect against the cognitive deficits that come from poor sleep.

However, Lambiase et al. (2014) and Wilckens et al. (2018) used *behavioral* measures of physical activity rather than *physiological* measures of activity, i.e., cardiorespiratory fitness. Although cardiorespiratory fitness and physical activity are related, the two may diverge depending on intensity and duration of the activity that is being performed. For example, engaging in bursts of high-intensity activity for a short period of time can produce greater increases in fitness than engaging in a lighter exercise for longer periods of time (Ramos et al., 2015). Cardiorespiratory fitness may be a key factor in the buffering the effects of poor sleep on cognition given that higher fit adults not only sleep better (Shapiro et al., 1984) but they also function better cognitively (Barnes et al., 2003) and are at a reduce risk of cognitive decline and dementia (Nyberg et al., 2014). Furthermore, these two studies provide promising evidence for an exercise-by-sleep interaction that promotes EF. It remains unclear whether this same relationship holds true for memory. Theoretically, physical exercise should protect memory in poor sleepers, as has been shown in animal models (Zagaar et al., 2013a,b; Zagaar, 2019), but this has yet to be examined in humans.

The present study sought to fill these two gaps by examining the interaction between cardiorespiratory fitness, sleep, and memory. Based off results of Lambiase et al. (2014) we hypothesized that cardiorespiratory fitness would moderate the relationship between sleep and memory in OA, suggesting that cardiorespiratory fitness is neuroprotective. Specifically, we hypothesized that OA with poor sleep would have worse memory if they were low fit, but not if they were high fit. We also tested this in YA but expected a weaker association.

## Materials and Methods

### Setting

This study took place at McMaster University in Hamilton, Ontario. Participant recruitment began in August 2019, and data collection took place between September 2019 to March 2020. Data collection ended prior to the COVID-19 lockdowns. Participants were recruited through posters and advertisements in local news outlets, posted throughout the Hamilton community and on McMaster University campus. Participants were also recruited from a participant database consisting of participants who have previously completed studies in the NeuroFit Lab or through McMaster’s Department of Psychology, Neuroscience, and Behavior Research participation system (SONA).

### Participants

The present study was part of larger, unpublished master’s thesis. In this thesis, the sample size estimate was calculated using G*Power (Version 3.1.9.3; Faul et al., 2007), based on the age differences (20–39 years old versus 60–74 years old) in mean delayed verbal memory scores (*d* = 0.71) found in Stark et al. (2013). Using the parameters of power being 0.90 and alpha equaling 0.05, G*Power indicated a total of 86 participants would be required: 43 YA and 43 OA. In that same study by Stark et al. (2013), they found a negative correlation between age and high-interference memory (*r* = −0.48), which converts to a Cohen’s *d* of 1.00 (Ruscio, 2008), suggesting that a total sample size of 46 would have adequate power to detect differences in mean high-interference memory scores between YA and OA.

The study was conducted between September 2019 and March 2020. Prior to the onset of the global COVID-19 pandemic, a total of 73 participants were recruited (YA, *n* = 44; OA, *n* = 29). Three participants did not complete both visits, due to ineligibility requirements (OA, *n* = 1), scheduling conflicts (YA, *n* = 1), or complications due to COVID-19 (YA, *n* = 1). Additionally, seven participants (YA, *n* = 5; OA, *n* = 2) had been recruited and scheduled but could not participate due to COVID-19 restriction on human research. In total 63 participants completed the study (YA, *n* = 37; OA, *n* = 26).

Participants were eligible to participate if they were between the ages of 18–30 or 65–79 and free from diagnosis of cognitive impairment, sleep apnea, psychiatric and neurological conditions, and were non-smokers, not obese [class I, body mass index (BMI) < 35], not taking hormone replacement therapy or beta-blockers. Additionally, participants were required to have normal sleep patterns with a regular sleep phase between 9:00 p.m. and 10:00 a.m. (Finan et al., 2019). Fulfillment of these criteria was confirmed verbally or written, either by email or over the phone. Prior to their first visit, eligible OA were required to obtain written consent from their physician to participate in a sub-maximal cardiorespiratory fitness assessment. Participants were also screened for cognitive impairment using the Montreal Cognitive Assessment (MoCA; Nasreddine et al., 2005). A normal score is considered to be ≥26, and a score of <23 is a suggested cut-off that may differentiate healthy cognition from cognitive impairment (Carson et al., 2018). The MoCA has good reliability (Cronbach’s alpha = 0.83), and good sensitivity and specificity for detecting MCI (90%) and AD (100%), and good specificity (87%) (Nasreddine et al., 2005).

All participants provided informed consent upon the first visit and were compensated with either $30 or three SONA credits for their participation. This study received ethics clearance from the McMaster Research Ethics Board (MREB #2079).

### Cardiorespiratory Fitness

Cardiorespiratory fitness (i.e., VO_2_ peak) was estimated using the Rockport 1-mile walk test (Kline et al., 1987). The Rockport 1-mile walk test has been validated in adults, and correlates highly with traditional treadmill tests to assess cardiorespiratory fitness (Kline et al., 1987; Colcombe et al., 2003, 2004). Participants were instructed to walk one mile as fast as they could, without running or powerwalking. Two trained research assistants supervised the test: one member of the research team recorded heart rate (using Polar FT1 heart rate monitors) at one-minute intervals and upon completion, while the second research assistant recorded distance using a surveyor’s wheel. Participants completed the Rockport 1-mile walk test on an indoor track located in the Physical Activity Center of Excellence at McMaster University. The following equation was used to estimated VO_2_ peak (Rockport Shoes Walking Institute, 1986):


$$\mathrm{Estimated}{\mathrm{VO}}_{2}\max\mathrm{ml}\cdot {\mathrm{kg}}^{-1}\cdot \min^{-1}=132.853-0.0769\left(\mathrm{weight}\mathrm{in}\mathrm{pounds}\right)-0.3877\left(\mathrm{age}\mathrm{in}\mathrm{years}\right)+6.315\left(\mathrm{if}\mathrm{male}\right)-3.2649\left(\mathrm{time}\mathrm{in}\mathrm{minutes}\right)-0.1565\left(\mathrm{final}\mathrm{heart}\mathrm{Rate}\right)$$

### Memory

The Mnemonic Similarities Task set C (MST) was used to measure high-interference memory and general recognition memory (Stark et al., 2013; Stark and Stark, 2017). High-interference memory is a type of hippocampus-dependent memory that helps one discern between highly similar, but different events. High-interference memory (also referred to as pattern separation) is associated with activity in the dentate gyrus (Yassa and Stark, 2011), a structure of the hippocampus where neurogenesis occurs (Zhao et al., 2008). Additionally, this type of memory is influenced by both cardiorespiratory fitness (Bullock et al., 2018) and sleep (Saletin et al., 2016). General recognition memory reflects the ability to discriminate new stimuli from previously observed stimuli (Mandler, 1980). General recognition memory is thought to depend on frontal and parietal brain regions (Neufang et al., 2006), making it less depend on hippocampal neurogenesis (Yonelinas et al., 2005) and less susceptive to changes in aging (Stark et al., 2013; Bullock et al., 2018).

**FIGURE 1:**
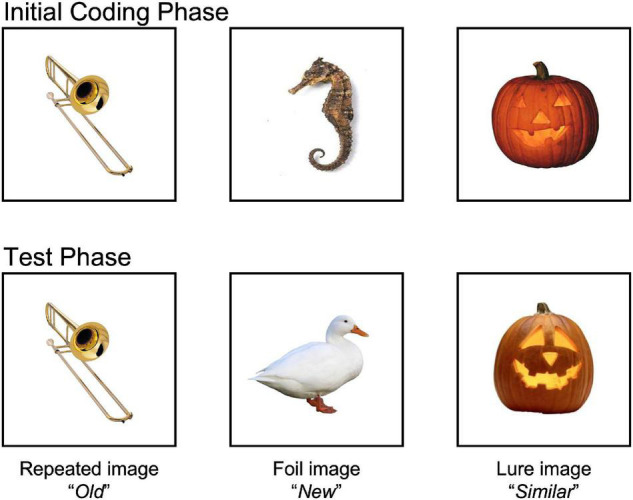
Mnemonic Similarities Task (MST). The MST measures hippocampal dependent memory by having participants discriminate between highly similar, but different memory. During the initial coding phase, participants were presented with 60 images that they had to identify as “indoor” or “outdoor” objects. During the test phase, participants were presented with 192 additional images. A total of 64 images were exact repetitions (images presented during the initial coding phase; correct response = “Old”), 64 novel foils (new images they had not been seen before; correct response = “New”), and 64 images were similar lures (images that were similar to a previously shown image, but not exactly identical; correct response = “similar”). High-interference memory was calculated by subtracting the proportion of *lures* that were correctly identified as “Similar” from the proportion of *foils* that were mistakenly identified as “Similar” [p(“Similar”| Lure) – p(“Similar”| Foil)]. This corrects for any overall bias of responding “Similar” to stimuli. General recognition memory was determined by whether participants correctly identified *repeated stimuli* as “Old” from the proportion of *foils* that were mistakenly identified as “Old” [p(“Old”| Repeat) – p(“Old”| Foil)]. Images available at: https://faculty.sites.uci.edu/starklab/mnemonic-similarity-task-mst/.

See Figure 1 for a visual representation of the MST task. During the MST, there is an incidental coding phase where participants were shown 60 full colored images, presented on the screen for 2 s. A blank screen preceded each trial for 500 ms. Participants were instructed to classify items as indoor, pressing the “N” key, or outdoor, pressing the “V” key. After this phase of the task, participants watched a video with instructions for the test phase of the task. During the test phase, participants were shown more images that they had to classify as either “Old” (repetitions), “Similar” (lures), or “New” (foils) using the “V” key, the “B” key and the “N” key, respectively. The test phase consisted of 192 items in total: 64 repetitions, 64 lures, 64, foils. Items were presented for 2.5 s, followed by a blank screen.

High-interference memory was calculated by subtracting the proportion of *lures* that were correctly identified as “Similar” from the proportion of *foils* that were mistakenly identified as “Similar” [p(“Similar”| Lure) – p(“Similar”| Foil)] (Stark et al., 2013; Stark and Stark, 2017). By doing so, this corrects for any overall bias of responding “Similar” to stimuli. Likewise, general recognition memory was determined by subtracting the proportion of correctly identified *repeated stimuli* as “Old” from the proportion of *foils* that were mistakenly identified as “Old” [p(“Old”| Repeat) – p(“Old”| Foil)]. Data was screened to ensure task comprehension. If participants did not use all keys, it was thought they did not understand the task, and would be removed from all memory analyses.

### Sleep

#### Actigraphy

Actigraphy was used as the primary sleep measure using the CenterPoint Insight watch (ActiGraph, LLC, Pensacola, FL, United States). Sleep periods were determined using the Cole-Kripke algorithm (Cole et al., 1992), which measures sleep using the *y*-axis epochs over a 7-min period to determine wake from sleep. A trained research assistant uploaded, compared, and adjusted sleep periods for all participants based on sleep diary records to ensure restless sleep was not mistaken for wakefulness. Participants wore the actigraphy on their non-dominant hand, and participants were considered compliant if they wore the actigraph for at least five nights. The sleep measures examined were number of nighttime awakenings, sleep efficiency, wake after sleep onset (WASO), and total sleep time (TST). Average values between visits one and two were computed for each sleep variable.

#### Pittsburg Sleep Quality Index

As a secondary measure of sleep, the Pittsburgh Sleep Quality Index (PSQI) was used to assess subjective sleep quality (Buysse et al., 1989). This questionnaire asks participants about their sleep habits during the last month by looking at seven components: subjective sleep quality, sleep latency, sleep duration, sleep efficiency, difficulties sleeping, use of sleeping medications, and their sleepiness. Participants scores are categorized into severity/frequency of sleep disturbances (0 = least severe, 3 = most severe), for a maximum score of 21. A higher score in indicative of greater sleep disturbance, and a score of >5 indicates a poor sleeper (Buysse et al., 1989). The PSQI has a Cronbach’s alpha of 0.83, as well as good diagnostic sensitivity (89.6%) and specificity (86.5%) at distinguishing “good” versus “poor” sleepers (Buysse et al., 1989).

#### Sleep Journal

A sleep journal created by the National Sleep Foundation was used to measure and record participants bed and wake times. Participants were instructed to record each morning the time they went to bed, the time they roughly fell asleep, and the time they woke up that morning. These times were used to determine the in-bed and awake times for the actigraph measures, as restless sleep would be mistaken for awake time (Morgenthaler et al., 2007).

### Covariates

Covariates included age, biological sex, years of education, BMI and depression, as these variables relate to sleep (Pearson et al., 2006; Park et al., 2013; Mallampalli and Carter, 2014; Moraes et al., 2014; Mander et al., 2017) and/or cognition (Hammar and Årdal, 2009; Salthouse, 2019; Lövdén et al., 2020). Age, biological sex, and years of education were assessed *via* demographics questionnaire. Depression was assessed using the Beck Depression Inventory (BDI; Beck et al., 1996). This is a 21-item questionnaire, requiring participants to select which statement best describes who they have been feeling during the previous 2 weeks, with responses ranging in intensity. A minimum score is 0 and a maximum score is 63. The BDI has a Cronbach’s alpha of 0.91 (Dozois et al., 1998) in YA and 0.86 in OA (Segal et al., 2008), and has good convergent and discriminant validity in both age groups (Segal et al., 2008).

### Procedure

This study was completed over the course of two visits. During the first visit, anthropometric measurements were taken, including weight and height, which were used to calculate BMI. Participants completed the MoCA to screen for cognitive impairment, and then performed the Rockport 1-Mile walk test. Upon completion, participants returned to the lab and filled out a demographic questionnaire. Participants were then given an actigraph and instructed to wear at all times for 1 week, except during bathing or swimming activities. Participants were also given a sleep journal to record their sleep over the week.

Following 1 week of tracking, participants returned to the lab and performed the MST. Finally, participants filled out the BDI and were debriefed.

### Statistical Analysis

Data was analyzed using *R* 4.0.5 programming software (R Core Team, 2021). Data was screened for extreme outliers, where values beyond < Q1 – 3*IQR or > Q3 + 3*IQR were removed. Descriptive statics were calculated for all study variables. Normality was assessed using Shapiro–Wilk tests and through visual inspection of histograms. Normality of residuals was inspected using Q–Q plots. Independence was measured using Durbin-Watson tests. Influential cases were screened using Cook’s distance. For all statistical analysis, two-tailed tests were used, and significance was considered at *p* < 0.05. Covariates included sex, years of education, BMI, age, and depression.

To test the moderating effect of cardiorespiratory fitness on the relationship between sleep and memory, multiple linear regressions were used to examine the interaction between cardiorespiratory fitness and sleep variables. Within each age group and stratified by sex, participants were categorized as “high fit” or “low fit” *via* median split, like the methodology used by Lambiase et al. (2014). If there was a significant interaction, the simple slopes for “high fit” and “low fit” in the model were calculated using the *reghelper* package (Hughes, 2021). These analyses were done separately for YA and OA. A baseline model that included only the covariates was used to calculate Δ*R*^2^.

## Results

### Data Screening and Assumptions

A total of 63 participants (OA, *n* = 26; YA, *n* = 37) completed both visits in the study. Two participants were removed from all analysis due to low actigraph compliance (wearing <5 nights; YA, *n* = 2). The final sample consists of 61 participants (OA, *n* = 26; YA, *n* = 35).

The data was screen for missing data; 0.68% of the data was missing. Two participants failed to properly fill out the PSQI (YA, *n* = 1; OA, *n* = 1), so their PSQI measures were removed, but their actigraphy data remained included. High-interference memory scores were removed if participants failed to understand the task, as indicated by failing to press all three keys (OA, *n* = 1). Two participants had extreme scores for their recognition memory scores (YA, *n* = 1; OA, *n* = 1), removed from their respective analyses.

All data met the assumption of normality using the Shapiro–Wilk test and visual inspection of histograms. All linear regression assumptions were met.

### Descriptive Statistics

Descriptive characteristics of the sample stratified by cardiorespiratory fitness levels are presented in Table 1. YA were aged 18–30, mostly female (24/35), and most were categorized as good sleepers (23/34). Older adults were aged 66–76 years old; half were female (13/26), most were categorized as good sleepers by the PSQI (18/25), and all were well educated (23/26 having >12 years of education). Overall, OA had lower fitness, higher BMI, and lower levels of depression compared to YA. Older adults also performed worse on tests of high-interference memory but had similar MoCA scores, general recognition memory scores, and years of education compared to YA. YA slept worse than OA, in that YA had more nighttime awakenings than OA [*t*(52.82) = 2.65, *p* = 0.011, *d* = 0.51]. Sleep duration was also significantly shorter in YA than in OA [*t*(55.81) = −2.08, *p* = 0.042, *d* = 0.83]. No significant differences were found for sleep efficiency or PSQI scores.

**TABLE 1:** Descriptive characteristics of the present sample.

	Young adults		Older adults	
	Lower fit	Higher fit	Lower fit	Higher fit
*N*	18	17	12	14
AGE (years)	21.50 (3.65)	20.35 (2.37)	71.4 (3.03)	70.14 (2.56)
EDUCATION (years)	17.72 (2.42)	16.94 (2.66)	17.91 (3.09)	17.54 (3.19)
BMI	23.39 (4.07)	21.66 (2.47)	27.3 (3.77)	24.28(2.33)*
MOCA	26.94 (1.06)	26.94 (2.05)	27 (1.95)	27.07 (2.23)
BDI	8.56 (6.47)	6.70 (5.33)	3.00 (1.60)	3.38 (4.01)
High-interference memory (% correct)	39.95 (14.05)	21.18 (24.44)	21.18 (24.44)	10.19 (16.42)
GENERAL RECOGNITION MEMORY	82.78 (7.19)	80.69 (9.53)	81.5 (8.33)	78.50 (12.23)
ESTIMATED VO_2_	42.63 (5.20)	49.40***(3.89)	21.30 (5.20)	27.76(4.12)**
NIGHTTIME AWAKENINGS (NUMBER OF AWAKENINGS)	18.98 (5.24)	19.89 (6.35)	15.05 (5.08)	15.69 (6.82)
SLEEP EFFICIENCY (%)	90.43 (3.54)	88.04 (4.43)	88.42 (3.95)	90.40 (3.35)
TST (minutes)	433.10 (52.61)	453.59 (54.31)	453.59 (54.31)	442.52 (31.65)
WASO	45.75 (14.22)	60.50 (23.49)	60.50 (23.49)	47.76 (19.77)
PSQI global score	4.94 (1.66)	4.44 (1.90)	4.83 (2.29)	4.00 (2.68)

BMI, body mass index; MoCA, Montreal Cognitive Assessment; BDI, Beck Depression Inventory; TST, total sleep time; WASO, wake after sleep onset; PSQI, Pittsburgh Sleep Quality Index. p-Value denotes results of independent t-test comparing high fit to low fit adults in their respective age groups. Sleep variables reflect the average values between visit one and two. *p < 0.05, **p < 0.01, ***p < 0.001.

When comparing between cardiorespiratory fitness levels (Table 1), as expected, higher fit adults had higher cardiorespiratory fitness in both YA and OA. Furthermore, higher fit adults had lower BMI, but this was only observed for OA. No other group differences were observed.

### The Moderating Effect of Cardiorespiratory Fitness on Sleep and Memory

**FIGURE 2:**
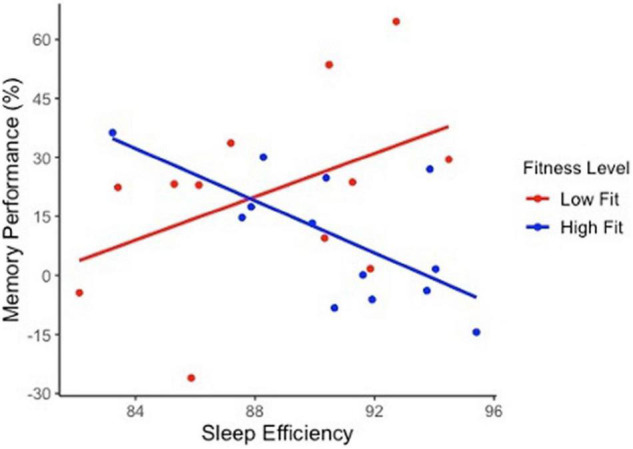
The moderating effect of cardiorespiratory fitness on sleep quality and high-interference memory performance in older adults. Sleep efficiency significantly predict cognitive performance in the lower fit group (*b* = 3.77, *SE b* = 1.65, *p* = 0.037) but not in higher fit group (*b* = –2.81, *SE b* = 1.71, *p* = 0.12). Trend lines are not adjusted to covariates.

Table 2 presents regression values for the moderating effect of cardiorespiratory fitness on sleep and high-interference memory in OA. Cardiorespiratory fitness significantly moderated that relationship between high-interference memory performance with nighttime awakenings, sleep efficiency and WASO. Three findings emerged. First, low fit OA with more nighttime awakenings had poorer high-interference memory (*b* = −3.94, *SE b* = 1.12, *p* = 0.0018). This same negative association between nighttime awakening and memory was not observed in high fit OA, but instead a positive association was present (*b* = 1.56, *SE b* = 0.69, *p* = 0.04). Second, low fit OA who slept more efficiently had better high-interference memory (*b* = 3.77, *SE b* = 1.65, *p* = 0.037) Figure 2. Again, this same positive association between sleep efficiency and memory was not observed in high fit OA (*b* = −2.81, *SE b* = 1.71, *p* = 0.12). Finally, there was no significant relationship between WASO and memory performance in both low fit (*b* = −0.49, *SE b* = 0.28, *p* = 0.09) and high fit (*b* = 0.48, *SE b* = 0.29, *p* = 0.12) OA. There was no significant moderating effect of cardiorespiratory fitness and sleep on general recognition memory in OA.

**TABLE 2:** Regression coefficients of the moderating effect of cardiorespiratory fitness and sleep on high-interference memory, in older adults.

	Δ *R*^2^	*b*	*SE b*	95% CIs	*p*
	0.43				**0.0047**
Nighttime awakenings		–3.94	1.05	(−6.17, −1.70)	**0.0118**
Estimated VO_2_		–93.10	21.40	(−138.47, −47.73)	**0.0005**
Interaction		5.50	1.31	(2.72, 8.28)	**0.0007**
	0.27				0.068
Sleep efficiency		3.77	1.65	(0.263, 7.28)	**0.037**
Estimated VO_2_		586.34	221.37	(117.06, 1055.62)	**0.018**
Interaction		–6.58	2.44	(−11.75, −1.42)	**0.016**
	0.23				0.13
WASO		–0.49	0.28	(−1.076, 0.103)	0.099
Estimated VO_2_		–56.32	21.93	(−102.80, −9.83)	**0.021**
Interaction		0.96	0.41	(0.10, 1.82)	**0.031**
	0.12				0.42
TST		0.08	0.12	(−0.18, 0.34)	0.54
Estimated VO_2_		–83.25	105.88	(−307.69, 141.20)	0.44
Interaction		0.17	0.23	(−0.33, 0.66)	0.48
	0.28				0.09
PSQI – global score		2.40	2.85	(−3.68, 8.48)	0.41
Estimated VO_2_		22.92	18.59	(−16.71, 62.54)	0.24
Interaction		–6.85	–1.97	(−14.27, 0.56)	0.067

*WASO, wake after sleep onset; TST, total sleep time; PSQI, Pittsburgh Sleep Quality Index. In all moderation models, cardiorespiratory fitness (estimated VO_2_) served as the moderator, sleep acted as the independent variable, and memory performance acted the dependent variable. Covariates included sex, age, BMI, depression, and years of education. Bold values were to indicate that these were statistically significant (P < 0.05).*

Table 3 presents regression values for the moderating effect of cardiorespiratory fitness on sleep and high-interference memory in YA. In contrast to OA, there were no significant interactions between cardiorespiratory fitness and sleep with high-interference memory or general recognition memory in YA.

**TABLE 3:** Regression coefficients of the moderating effect of cardiorespiratory fitness and sleep on high-interference memory, in young adults.

	Δ *R*^2^	*b*	*SE b*	95% CIs	*p*
	0.10				0.35
Nighttime awakenings		–0.65	1.00	(−2.71, 1.4)	0.53
Estimated VO_2_		13.22	21.48	(−30.94, 57.4)	0.54
Interaction		–0.17	1.190	(−2.44, 2.1)	0.88
	0.07				0.53
Sleep efficiency		0.90	1.85	(−2.9, 4.7)	0.63
Estimated VO_2_		73.39	156.50	(−248.31, 395.09)	0.64
Interaction		–0.71	1.73	(−4.25, 2.84)	0.69
	0.07				0.51
WASO		–0.14	0.48	(−1.13, 0.84)	0.77
Estimated VO_2_		–1.62	20.32	(−43.41, 40.17)	0.94
Interaction		0.48	0.23	(−0.71, 1.13)	0.64
	0.17				0.12
TST		0.08	0.09	(−0.10, 0.26)	0.37
Estimated VO_2_		–86.34	71.99	(−234.32, 61.64)	0.24
Interaction		0.23	0.17	(−0.12, 0.58)	0.19
	0.11				0.52
PSQI – global score		2.29	2.60	(−3.07, 7.7)	0.39
Estimated VO_2_		20.04	18.38	(−17.81, 57.9)	0.29
Interaction		–2.71	3.77	(−10.47, 5.0)	0.48

*WASO, wake after sleep onset; TST, total sleep time; PSQI, Pittsburgh Sleep Quality Index. In all moderation models, cardiorespiratory fitness (estimated VO_2_) served as the moderator, sleep acted as the independent variable, and memory performance acted the dependent variable. Covariates included sex, age, BMI, depression, and years of education.*

## Discussion

The objective of this study was to examine the moderating effect of cardiorespiratory fitness on sleep and memory. We hypothesized that OA with poor sleep would have worse memory if they were low fit, but not if they were high fit; thus, pointing to the neuroprotective role of cardiorespiratory fitness in aging. Indeed, this is what we observed. Among low fit OA, high-interference memory performance was directly related to their sleep quality, such that every nighttime awakening was associated with a ∼4% decrease in high-interference memory; likewise, every 1% decrease in sleep efficiency was associated with a ∼4% decrease in high-interference memory. Critically these results were only observed for low fit OA and not for high fit OA suggesting that higher fitness in OA may protect against memory impairment from poor sleep.

Notably, we only observed associations for high-interference memory and not for general recognition memory. A critical difference between these two types of memory is that high-interference memory is more dependent on hippocampal structures (Yassa and Stark, 2011), than recognition memory (Yonelinas et al., 2005; Neufang et al., 2006), because of this, high-interference memory may be more dependent on hippocampus neurogenesis, which declines with age (Kuhn et al., 1996; Apple et al., 2017) and increases with exercise (Van Praag et al., 1999; Creer et al., 2010). In the context of the present study, these prior results suggest that high fitness in OA may protect against hippocampal-related memory impairment from poor sleep, as was observed here. Though it should be noted that the results observed here for high-interference memory are similar those found by Lambiase et al. (2014), who examined physical activity levels are EF, suggesting this relationship may exist in other aspects of cognition.

Why might sleep quality relate to high-interference memory performance? To answer this question, it is important to examine the aspect of sleep affected. Here, we found that poorer memory in OA was associated with greater nighttime awakenings and low sleep efficiency, but not WASO. This suggests that the frequency of sleep disturbance (i.e., the number of nighttime awakenings) may have a greater impact on cognitive functioning than the duration of time spent awake during these disturbances (i.e., the total duration of WASO) (Bonnet, 1986). This may reflect a decrease in the opportunity for OA to enter SWS, which is the most restorative aspect of sleep that is important for the processing memories (Walker, 2009). While diminishing SWS is often reported with aging (Ohayon et al., 2004; Moraes et al., 2014; Mander et al., 2017), SWS is also reduced in individuals with sleep apnea, who often experienced disrupted sleep due to nighttime cessations in breathing (Redline et al., 2004; Peregrim et al., 2019; Ren et al., 2020) and are at an increased their risk of dementia. That is because SWS plays a critical role in preventing the accumulation of beta-amyloid (Xie et al., 2013; Fultz et al., 2019; Ju et al., 2019)—a hallmark pathology in Alzheimer’s disease (Lucey and Bateman, 2014; Lucey, 2020).

An important implication of these findings is that low fit individuals may be able to improve their memory by improving their sleep quality. This is good news for people who are unable to be sufficiently active for good health due to pain, risk of falls, or other mobility limitations. Although physical activity is one way to improve sleep quality (Kredlow et al., 2015; Dolezal et al., 2017), it is not the only way. Other lifestyle interventions such as cognitive behavioral therapy or mindfulness practice have been shown to improve sleep in OA (MacLeod et al., 2018). In combination with these practices, engaging in other habits, behaviors, and environmental factors that promote sleep can also improve OA’ sleep hygiene and overall quality of sleep (MacLeod et al., 2018).

Interestingly, we did not see this same relationship between good sleep and memory in low fit YA, suggesting that the neuroprotective effects of sleep and fitness may be more pronounced later in life. This may not be too surprising given that YA are likely at the peak of their cognitive abilities, including stronger memory and hippocampal integrity compared to OA (Raz et al., 2005, 2010; Salthouse, 2019); their robust brain functioning may be less affected by lifestyle (Bullock et al., 2018). This seems to be especially true when examined cross-sectionally as done here. In contrast, an acute bout of exercise can alter cognition in YA but often elicits only immediate effects in the acute phase following exercise, when neurotrophic factors like brain-derived neurotrophic factor (BDNF) peak (Etnier et al., 2016; García-Suárez et al., 2021). BDNF promotes hippocampal neuroplasticity (Erickson et al., 2011; Ruscheweyh et al., 2011; Leckie et al., 2014), and exercise helps staves off the typically decline in neuroplasticity and BDNF that is associated with aging and poor sleep. Interestingly, animal models show that rodents who engage in regularly physical activity maintain high levels of BDNF despite being sleep deprived, and this protects them from the expected poor sleep-related memory impairment (Zagaar et al., 2013a,b; Zagaar, 2019). Taken together, these results suggests that fitness-related benefits on the brain may help counteract the typical deleterious effects of aging and poor sleep on memory.

### Strengths and Limitations

The use of actigraphy provides mixed strengths and limitations to this study. It is a more accurate and reliable measure of sleep than subjective measurements of sleep, such as the PSQI, which are often uncorrelated with objective measures of sleep (Buysse et al., 2008) and highly dependent on one’s mental health status (Buysse et al., 2008; Dietch et al., 2016). Another strength of actigraphy is that it can measure sleep naturalistically and over an extended period. A limitation of actigraphy is it can only detect the differences between wakefulness and sleep but cannot capture aspects of sleep architecture like SWS.

Another limitation of the study is its small sample size; unfortunately, the study was forced to end prematurely due to the COVID-19 pandemic restriction on human research. Nevertheless, it is worth noting that the significant relationships observed here ranged from medium (*f*^2^ = 0.14) to large (*f*^2^ = 0.75) (Cohen, 1988).

## Conclusion

In conclusion, the present study suggests that sleep and cardiorespiratory fitness may interact to enhance memory, and that this effect may be specific to OA. While poor sleep was associated with worse memory in low fit OA, the detriment of poor sleep on memory was negated (and even enhanced) in OA who were high fit. These results suggest cardiorespiratory fitness may protect OA from sleep-deficits in memory. Moreover, some low fit OA had good quality sleep which was associated with better memory performance, this suggesting OA who do not exercise may be able to promote their memory by focusing on other factors that promote good sleep. Finally, we did not see an association between cardiorespiratory fitness or sleep for cognition in YA, suggesting that the interplay between cardiorespiratory fitness, sleep and memory may be more pronounced as we get older.

## Statement of Significance

Aging is accompanied by a gradual decline in sleep. Poor sleep impairs cognition and may account for some of the age-related changes in memory. Exercise improves both sleep and cognition. Yet, very little work has examined the interplay between the three. Our results suggest that physical fitness may protect older adults’ memory from poorer sleep. Lower fit older adults who had poorer sleep also had worse memory, but memory was improved with better sleep. In contrast, high fit older adults who slept poorly still had good memory. The effect was exclusive to older adults, and not seen in younger adults. These results have important implications for research and clinicians interested in lifestyle approaches that promote cognition across the lifespan, as these results suggest that living a physically active lifestyle may counteract the deleterious effects of poor sleep on memory. Additionally, it also suggests that in adults who do not or cannot be physically active, finding ways to promote good sleep quality may help protect their memory, such as engaging in cognitive behavioral therapy or promoting sleep hygiene. In all, these results show lifestyle factors interact with one another to promote healthy cognitive aging.

## Data Availability Statement

The raw data supporting the conclusions of this article will be made available by the authors, without undue reservation.

## Ethics Statement

The studies involving human participants were reviewed and approved by the McMaster Research Ethics Board (MREB). The patients/participants provided their written informed consent to participate in this study.

## Author Contributions

TK led the study design, data collection, data analyses, interpretation of the data, and drafted the manuscript. JH secured the funding, contributed to the advising of the study design, interpreting results, and revising the manuscript. Both authors contributed to the article and approved the submitted version.

## Conflict of Interest

The authors declare that the research was conducted in the absence of any commercial or financial relationships that could be construed as a potential conflict of interest.

## Publisher’s Note

All claims expressed in this article are solely those of the authors and do not necessarily represent those of their affiliated organizations, or those of the publisher, the editors and the reviewers. Any product that may be evaluated in this article, or claim that may be made by its manufacturer, is not guaranteed or endorsed by the publisher.

## References

[B1] Altena E. Ramautar J. R. Van Der Werf Y. D. Van Someren E. J. W. (2010). Do sleep complaints contribute to age-related cognitive decline? *Prog. Brain Res.* 185 181–205. 10.1016/B978-0-444-53702-7.00011-7 21075240

[B2] Apple D. M. Solano-Fonseca R. Kokovay E. (2017). Neurogenesis in the aging brain. *Biochem. Pharmacol.* 141 77–85. 10.1016/J.BCP.2017.06.116 28625813

[B3] Barnes D. E. Yaffe K. Satariano W. A. Tager I. B. (2003). A longitudinal study of cardiorespiratory fitness and cognitive function in healthy older adults. *J. Am. Geriatr. Soc.* 51 459–465. 10.1046/j.1532-5415.2003.51153.x 12657064

[B4] Beaulieu-Bonneau S. Hudon C. (2009). Sleep disturbances in older adults with mild cognitive impairment. *Int. Psychogeriatr.* 21 654–666. 10.1017/S1041610209009120 19426575

[B5] Beck A. T. Steer R. A. Brown G. K. (1996). *Manual For The Beck Depression Inventory-II.* San Antonio, TX: Psychological Corporation.

[B6] Blackwell T. Yaffe K. Ancoli-Israel S. Redline S. Ensrud K. E. Stefanick M. L. (2011). Association of sleep characteristics and cognition in older community-dwelling men: the MrOS sleep study. *Sleep* 34 1347–1356. 10.5665/SLEEP.1276 21966066 PMC3174836

[B7] Bonnet M. H. (1986). Performance and sleepiness as a function of frequency and placement of sleep disruption. *Psychophysiology* 23 263–271. 10.1111/j.1469-8986.1986.tb00630.x 3749406

[B8] Bruce A. S. Aloia M. (2006). Sleep and Cognition in Older Adults. *Sleep Med. Clin.* 1 207–220. 10.1016/J.JSMC.2006.04.008

[B9] Bullock A. M. Mizzi A. L. Kovacevic A. Heisz J. J. (2018). The association of aging and aerobic fitness with memory. *Front. Aging Neurosci.* 10:63. 10.3389/fnagi.2018.00063 29593524 PMC5854680

[B10] Buysse D. J. Hall M. L. Strollo P. J. Kamarck T. W. Owens J. Lee L. (2008). Relationships between the Pittsburgh Sleep Quality Index (PSQI), Epworth Sleepiness Scale (ESS), and clinical/polysomnographic measures in a community sample. *J. Clin. Sleep Med.* 4 563–571. 10.5664/jcsm.27351 19110886 PMC2603534

[B11] Buysse D. J. Reynolds C. F. Monk T. H. Berman S. R. Kupfer D. J. (1989). The Pittsburgh sleep quality index: a new instrument for psychiatric practice and research. *Psychiatry Res.* 28 193–213. 10.1016/0165-1781(89)90047-4 2748771

[B12] Carson N. Leach L. Murphy K. J. (2018). A re-examination of Montreal Cognitive Assessment (MoCA) cutoff scores. *Int. J. Geriatric Psychiatry* 33 379–388. 10.1002/gps.4756 28731508

[B13] Chang M. Jonsson P. V. Snaedal J. Bjornsson S. Saczynski J. S. Aspelund T. (2010). The effect of midlife physical activity on cognitive function among older adults: AGES - Reykjavik study. *J. Gerontol. A Biol. Sci. Med. Sci.* 65 1369–1374. 10.1093/gerona/glq152 20805238 PMC2990266

[B14] Chaput J. P. Wong S. L. Michaud I. (2017). Duration and quality of sleep among Canadians aged 18 to 79. *Health Rep.* 28 28–33. 28930365

[B15] Cohen J. (1988). *Statistical Power Analysis For The Behavioral Sciences.* Hillsdale, NJ: Lawrence Erlbaum Associates, Inc.

[B16] Colcombe S. J. Erickson K. I. Raz N. Webb A. G. Cohen N. J. McAuley E. (2003). Aerobic fitness reduces brain tissue loss in aging humans. *J. Gerontol. Ser. A Biol. Sci. Med. Sci.* 58 M176–M180. 10.1093/gerona/58.2.m176 12586857

[B17] Colcombe S. J. Kramer A. F. Erickson K. I. Scalf P. McAuley E. Cohen N. J. (2004). Cardiovascular fitness, cortical plasticity, and aging. *Proc. Natl. Acad. Sci. U.S.A.* 101 3316–3321. 10.1073/pnas.0400266101 14978288 PMC373255

[B18] Colcombe S. Kramer A. F. (2003). Fitness effects on the cognitive function of older adults. *Psychol. Sci.* 14 125–130. 10.1111/1467-9280.t01-1-01430 12661673

[B19] Cole R. J. Kripke D. F. Gruen W. Mullaney D. J. Gillin J. C. (1992). Automatic sleep/wake identification from wrist activity. *Sleep* 15 461–469. 10.1093/sleep/15.5.461 1455130

[B20] Creer D. J. Romberg C. Saksida L. M. Van Praag H. Bussey T. J. (2010). Running enhances spatial pattern separation in mice. *Proc. Natl. Acad. Sci. U.S.A.* 107 2367–2372. 10.1073/pnas.0911725107 20133882 PMC2836679

[B21] Diamond A. (2013). Executive functions. *Ann. Rev. Psychol.* 64 135–168. 10.1146/annurev-psych-113011-143750 23020641 PMC4084861

[B22] Dietch J. R. Taylor D. J. Sethi K. Kelly K. Bramoweth A. D. Roane B. M. (2016). Psychometric evaluation of the PSQI in U.S. college students. *J. Clin. Sleep Med.* 12 1121–1129. 10.5664/jcsm.6050 27166299 PMC4957190

[B23] Dolezal B. A. Neufeld E. V. Boland D. M. Martin J. L. Cooper C. B. (2017). Interrelationship between sleep and exercise: a systematic review. *Adv. Prev. Med.* 2017 1–14. 10.1155/2017/1364387 28458924 PMC5385214

[B24] Dozois D. J. A. Dobson K. S. Ahnberg J. L. (1998). A psychometric evaluation of the beck depression inventory-II. *Psychol. Assess.* 10 83–89. 10.1037/1040-3590.10.2.83

[B25] Erickson K. Voss M. Prakash R. S. Basak C. Szabo A. Chaddock L. (2011). Exercise training increases size of hippocampus and improves memory. *Proc. Natl. Acad. Sci.* 108 3017–3022. 10.1073/pnas.1015950108 21282661 PMC3041121

[B26] Etnier J. Wideman L. Labban J. Piepmeier A. Pendleton D. Dvorak K. (2016). The effects of acute exercise on memory and brain-derived neurotrophic factor (BDNF). *J. Sport Exerc. Psychol.* 38 331–340. 10.1123/JSEP.2015-0335 27385735

[B27] Faul F. Erdfelder E. Lang A.-G. Buchner A. (2007). G*Power 3: A flexible statistical power analysis program for the social, behavioral, and biomedical sciences. *Behav. Res. Methods* 39, 175–191. 10.3758/BF03193146 17695343

[B28] Finan P. H. Whitton A. E. Letzen J. E. Remeniuk B. Robinson M. L. Irwin M. R. (2019). Experimental sleep disruption and reward learning: moderating role of positive affect responses. *Sleep* 42 1–10. 10.1093/sleep/zsz026 30927744 PMC6519913

[B29] Fultz N. E. Bonmassar G. Setsompop K. Stickgold R. A. Rosen B. R. Polimeni J. R. (2019). Coupled electrophysiological, hemodynamic, and cerebrospinal fluid oscillations in human sleep. *Science* 366 628–631. 10.1126/science.aax5440 31672896 PMC7309589

[B30] García-Suárez P. C. Rentería I. Plaisance E. P. Moncada-Jiménez J. Jiménez-Maldonado A. (2021). The effects of interval training on peripheral brain derived neurotrophic factor (BDNF) in young adults: a systematic review and meta-analysis. *Sci. Rep.* 11:8937. 10.1038/s41598-021-88496-x 33903670 PMC8076263

[B31] Hammar Å Årdal G. (2009). Cognitive functioning in major depression-a summary. *Front. Hum. Neurosci.* 3:26. 10.3389/NEURO.09.026.2009 19826496 PMC2759342

[B32] Hirshkowitz M. Whiton K. Albert S. M. Alessi C. Bruni O. DonCarlos L. (2015). National sleep foundation’s updated sleep duration recommendations: final report. *Sleep Health* 1 233–243. 10.1016/j.sleh.2015.10.004 29073398

[B33] Hughes J. (2021). *reghelper: Helper Functions for Regression Analysis. R package Version 1.1.0.* Available online at: https://cran.r-project.org/web/packages/reghelper/index.html (accessed July 11, 2021).

[B34] Ju Y.-E. S. Zangrilli M. A. Finn M. B. Fagan A. M. Holtzman D. M. (2019). Obstructive sleep apnea treatment, slow wave activity, and amyloid-β. *Ann. Neurol.* 85:291. 10.1002/ANA.25408 30597615 PMC6396979

[B35] Kline G. M. Porcari J. P. Hintermeister R. Freedson P. S. Ward A. McCarron R. F. (1987). Estimation of VO2max from a one-mile track walk, gender, age, and body weight. *Med. Sci. Sports Exerc.* 19 253–259. 3600239

[B36] Kredlow M. A. Capozzoli M. C. Hearon B. A. Calkins A. W. Otto M. W. (2015). The effects of physical activity on sleep: a meta-analytic review. *J. Behav. Med.* 38 427–449. 10.1007/s10865-015-9617-6 25596964

[B37] Kubitz K. A. Landers D. M. Petruzzello S. J. Han M. (1996). The effects of acute and chronic exercise on sleep a meta-analytic review. *Sports Med.* 21 277–291. 10.2165/00007256-199621040-00004 8726346

[B38] Kuhn H. G. Dickinson-Anson H. Gage F. H. (1996). Neurogenesis in the dentate gyrus of the adult rat: age-related decrease of neuronal progenitor proliferation. *J. Neurosci.* 16 2027–2033. 10.1523/jneurosci.16-06-02027.1996 8604047 PMC6578509

[B39] Lambiase M. J. Gabriel K. P. Kuller L. H. Matthews K. A. (2014). Sleep and executive function in older women: the moderating effect of physical activity. *J. Gerontol. A Biol. Sci. Med. Sci.* 69 1170–1176. 10.1093/gerona/glu038 24744391 PMC4441058

[B40] Leckie R. L. Oberlin L. E. Voss M. W. Prakash R. S. Szabo-Reed A. Chaddock-Heyman L. (2014). BDNF mediates improvements in executive function following a 1-year exercise intervention. *Front. Hum. Neurosci.* 8:985. 10.3389/fnhum.2014.00985 25566019 PMC4263078

[B41] Lim A. S. P. Kowgier M. Yu L. Buchman A. S. Bennett D. A. (2013). Sleep fragmentation and the risk of incident alzheimer’s disease and cognitive decline in older persons. *Sleep* 36 1027–1032. 10.5665/sleep.2802 23814339 PMC3669060

[B42] Lim A. S. P. Yu L. Costa M. D. Leurgans S. E. Buchman A. S. Bennett D. A. (2012). Increased fragmentation of rest-activity patterns is associated with a characteristic pattern of cognitive impairment in older individuals. *Sleep* 35 633–40B. 10.5665/sleep.1820 22547889 PMC3321422

[B43] Loprinzi P. D. Frith E. Edwards M. K. Sng E. Ashpole N. (2018). The effects of exercise on memory function among young to middle-aged adults: systematic review and recommendations for future research. *Am. J. Health Promot.* 32 691–704. 10.1177/0890117117737409 29108442

[B44] Lövdén M. Fratiglioni L. Glymour M. M. Lindenberger U. Tucker-Drob E. M. (2020). Education and cognitive functioning across the life span. *Psychol. Sci. Public Interest* 21 6–41. 10.1177/1529100620920576 32772803 PMC7425377

[B45] Lucey B. P. (2020). It’s complicated: the relationship between sleep and Alzheimer’s disease in humans *Neurobiol. Dis.* 144, 1–8. 10.1016/j.nbd.2020.105031 32738506 PMC7484285

[B46] Lucey B. P. Bateman R. J. (2014). Amyloid-β diurnal pattern: possible role of sleep in Alzheimer’s disease pathogenesis *Neurobiol. Aging* 35 S29–S34. 10.1016/j.neurobiolaging.2014.03.035 24910393

[B47] MacLeod S. Musich S. Kraemer S. Wicker E. (2018). Practical non-pharmacological intervention approaches for sleep problems among older adults. *Geriatr. Nurs.* 39 506–512. 10.1016/J.GERINURSE.2018.02.002 29530293

[B48] Mallampalli M. P. Carter C. L. (2014). Exploring sex and gender differences in sleep health: a society for women’s health research report. *J. Womens Health* 23 553–562. 10.1089/jwh.2014.4816 24956068 PMC4089020

[B49] Mander B. A. Winer J. R. Walker M. P. (2017). Sleep and human aging. *Neuron* 94 19–36. 10.1016/j.neuron.2017.02.004 28384471 PMC5810920

[B50] Mandler G. (1980). Recognizing: the judgment of previous occurrence. *Psychol. Rev.* 87, 252–271. 10.1037/0033-295X.87.3.252

[B51] Middleton L. E. Barnes D. E. Lui L. Y. Yaffe K. (2010). Physical activity over the life course and its association with cognitive performance and impairment in old age. *J. Am. Geriatr. Soc.* 58 1322–1326. 10.1111/j.1532-5415.2010.02903.x 20609030 PMC3662219

[B52] Moraes W. Piovezan R. Poyares D. Bittencourt L. R. Santos-Silva R. Tufik S. (2014). Effects of aging on sleep structure throughout adulthood: a population-based study. *Sleep Med.* 15 401–409. 10.1016/j.sleep.2013.11.791 24657204

[B53] Morgenthaler T. Alessi C. Friedman L. Owens J. Kapur V. Boehlecke B. (2007). Practice parameters for the use of actigraphy in the assessment of sleep and sleep disorders: an update for 2007. *Sleep* 30 519–529. 10.1093/sleep/30.4.519 17520797

[B54] Nasreddine Z. S. Phillips N. A. BÃcdirian V. Charbonneau S. Whitehead V. Collin I. (2005). The montreal cognitive assessment, MoCA: a brief screening tool for mild cognitive impairment. *J. Am. Geriatr. Soc.* 53 695–699. 10.1111/j.1532-5415.2005.53221.x 15817019

[B55] Neufang M. Heinze H. J. Duzel E. (2006). Electromagnetic correlates of recognition memory processes. *Clin. EEG Neurosci.* 37, 300–308. 10.1177/155005940603700407 17073168

[B56] Nyberg J. Aberg M. A. I. Schiöler L. Nilsson M. Wallin A. Torén K. (2014). Cardiovascular and cognitive fitness at age 18 and risk of early-onset dementia. *Brain* 137 1514–1523. 10.1093/brain/awu041 24604561

[B57] Ohayon M. M. Carskadon M. A. Guilleminault C. Vitiello M. V. (2004). Meta-analysis of quantitative sleep parameters from childhood to old age in healthy individuals: developing normative sleep values across the human lifespan. *Sleep* 27 1255–1273. 10.1093/sleep/27.7.1255 15586779

[B58] Park J.-H. Yoo M.-S. Bae S. H. (2013). Prevalence and predictors of poor sleep quality in Korean older adults. *Int. J. Nurs. Pract.* 19 116–123. 10.1111/IJN.12047 23577968

[B59] Pearson N. Johnson L. Nahin R. (2006). Insomnia, trouble sleeping, and complementary and alternative medicine: analysis of the 2002 national health interview survey data. *Arch. Int. Med.* 166 1775–1782. 10.1001/ARCHINTE.166.16.1775 16983058

[B60] Peregrim I. Grešová S. Štimmelová J. Bačová I. Fulton B. Tokárová D. (2019). Strong coincidence between slow wave sleep and low AHI is explainable by the high instability of slow wave sleep to obstructive apnea exposure. *Physiol. Res.* 68 857–865. 10.33549/PHYSIOLRES.934025 31424253

[B61] R Core Team (2021). *R: A language and environment for statistical computing*. Vienna: R Foundation for Statistical Computing. Available online at: https://www.R-project.org/

[B62] Ramos J. S. Dalleck L. C. Tjonna A. E. Beetham K. S. Coombes J. S. (2015). The impact of high-intensity interval training versus moderate-intensity continuous training on vascular function: a systematic review and meta-analysis. *Sports Med.* 45 679–692. 10.1007/s40279-015-0321-z 25771785

[B63] Raz N. Ghisletta P. Rodrigue K. M. Kennedy K. M. Lindenberger U. (2010). Trajectories of brain aging in middle-aged and older adults: regional and individual differences. *Neuroimage* 51 501–511. 10.1016/j.neuroimage.2010.03.020 20298790 PMC2879584

[B64] Raz N. Lindenberger U. Rodrigue K. M. Kennedy K. M. Head D. Williamson A. (2005). Regional brain changes in aging healthy adults: general trends, individual differences and modifiers. *Cereb. Cortex* 15 1676–1689. 10.1093/cercor/bhi044 15703252

[B65] Redline S. Kirchner H. L. Quan S. F. Gottlieb D. J. Kapur V. Newman A. (2004). The effects of age, sex, ethnicity, and sleep-disordered breathing on sleep architecture. *Arch. Int. Med.* 164 406–418. 10.1001/ARCHINTE.164.4.406 14980992

[B66] Ren R. Covassin N. Zhang Y. Lei F. Yang L. Zhou J. (2020). Interaction between slow wave sleep and obstructive sleep apnea in prevalent hypertension. *Hypertension* 75 516–523. 10.1161/HYPERTENSIONAHA.119.13720 31865784

[B67] Rockport Shoes Walking Institute (1986). *Rockport One Mile Walking Test.* Malboro, MA: Rockport Walking Institute.

[B68] Roig M. Nordbrandt S. Geertsen S. S. Nielsen J. B. (2013). The effects of cardiovascular exercise on human memory: a review with meta-analysis. *Neurosci. Biobehav. Rev.* 37 1645–1666. 10.1016/j.neubiorev.2013.06.012 23806438

[B69] Ruscheweyh R. Willemer C. Krüger K. Duning T. Warnecke T. Sommer J. (2011). Physical activity and memory functions: an interventional study. *Neurobiol. Aging* 32 1304–1319. 10.1016/j.neurobiolaging.2009.08.001 19716631

[B70] Ruscio J. (2008). A probability-based measure of effect size: robustness to base rates and other factors. *Psychol. Methods* 13 19–30. 10.1037/1082-989X.13.1.19 18331151

[B71] Saletin J. M. Goldstein-Piekarski A. N. Greer S. M. Stark S. Stark C. E. Walker M. P. (2016). Human hippocampal structure: a novel biomarker predicting mnemonic vulnerability to, and recovery from, sleep deprivation. *J. Neurosci.* 36 2355–2363. 10.1523/JNEUROSCI.3466-15.2016 26911684 PMC4764658

[B72] Salthouse T. A. (2019). Trajectories of normal cognitive aging. *Psychol. Aging* 34 17–24. 10.1037/pag0000288 30211596 PMC6367038

[B73] Segal D. L. Coolidge F. L. Cahill B. S. O’Riley A. A. (2008). Psychometric properties of the beck depression inventory—II (BDI-II) among community-dwelling older adults. *Behav. Modif.* 32 3–20. 10.1177/0145445507303833 18096969

[B74] Shapiro C. M. Warren P. M. Trinder J. Paxton S. J. Oswald I. Flenley D. C. (1984). Fitness facilitates sleep. *Eur. J. Appl. Physiol. Occup. Physiol.* 53 1–4. 10.1007/BF00964680 6542495

[B75] Stark S. M. Stark C. E. L. (2017). Age-related deficits in the mnemonic similarity task for objects and scenes. *Behav. Brain Res.* 333 109–117. 10.1016/j.bbr.2017.06.049 28673769 PMC5760178

[B76] Stark S. M. Yassa M. A. Lacy J. W. Stark C. E. L. (2013). A task to assess behavioral pattern separation (BPS) in humans: data from healthy aging and mild cognitive impairment. *Neuropsychologia* 51 2442–2449. 10.1016/j.neuropsychologia.2012.12.014 23313292 PMC3675184

[B77] Sterniczuk R. Theou O. Rusak B. Rockwood K. (2013). Sleep disturbance is associated with incident dementia and mortality. *Curr. Alzheimer Res.* 10 767–775. 10.2174/15672050113109990134 23905991

[B78] Van Praag H. Kempermann G. Gage F. H. (1999). Running increases cell proliferation and neurogenesis in the adult mouse dentate gyrus. *Nat. Neurosci.* 2 266–270. 10.1038/6368 10195220

[B79] Walker M. P. (2009). The role of slow wave sleep in memory processing. *J. Clin. Sleep Med.* 5 S20–S26. 10.5664/jcsm.5.2S.S20 19998871 PMC2824214

[B80] Wilckens K. A. Erickson K. I. Wheeler M. E. (2018). Physical activity and cognition: a mediating role of efficient sleep. *Behav. Sleep Med.* 16 569–586. 10.1080/15402002.2016.1253013 27935322 PMC5466488

[B81] Xie L. Xie L. Kang H. Xu Q. Chen M. J. Liao Y. (2013). Sleep drives metabolite clearance from the adult brain. *Science* 342 373–377. 10.1126/science.1241224 24136970 PMC3880190

[B82] Yassa M. A. Stark C. E. L. (2011). Pattern separation in the hippocampus. *Trends Neurosci.* 34 515–525. 10.1016/j.tins.2011.06.006 21788086 PMC3183227

[B83] Yonelinas A. P. Otten L. J. Shaw R. N. Rugg M. D. (2005). Separating the brain regions involved in recollection and familiarity in recognition memory. *J. Neurosci.* 25, 3002–3008. 10.1523/JNEUROSCI.5295-04.2005 15772360 PMC6725129

[B84] Zagaar M. (2019). *Regular Treadmill Exercise Prevents Sleep Deprivation-Induced Impairment Of Hippocampal-Dependent Memory And Synaptic Plasticity*. Ph.D. thesis, University of Houston Institutional Repository. 10.1017/CBO9781107415324.004

[B85] Zagaar M. Dao A. Alhaider I. Alkadhi K. (2013a). Regular treadmill exercise prevents sleep deprivation-induced disruption of synaptic plasticity and associated signaling cascade in the dentate gyrus. *Mol. Cell. Neurosci.* 56 375–383. 10.1016/j.mcn.2013.07.011 23911794

[B86] Zagaar M. Dao A. Levine A. Alhaider I. Alkadhi K. (2013b). Regular exercise prevents sleep deprivation associated impairment of long-term memory and synaptic plasticity in the CA1 area of the hippocampus. *Sleep* 36 751–761. 10.5665/sleep.2642 23633758 PMC3624830

[B87] Zhao C. Deng W. Gage F. H. (2008). Mechanisms and functional implications of adult neurogenesis. *Cell* 132 645–660 10.1016/j.cell.2008.01.033 18295581

